# Effects of Bilayer Thickness on the Morphological, Optical, and Electrical Properties of Al_2_O_3_/ZnO Nanolaminates

**DOI:** 10.1186/s11671-017-2328-x

**Published:** 2017-10-11

**Authors:** Da-Hai Li, Chen-Hui Zhai, Wen-Chao Zhou, Qing-Hua Huang, Lei Wang, Hua Zheng, Lei Chen, Xin Chen, Rong-Jun Zhang

**Affiliations:** 1 Key Laboratory of Micro and Nano Photonic Structures, Ministry of Education, Department of Optical Science and Engineering, Fudan University, Shanghai, 200433 China; 2 State Key Laboratory of Applied Optics, Changchun Institute of Optics, Fine Mechanics and Physics, Chinese Academy of Sciences, Changchun, Jilin 130033 China; 3 Institute of Electronic Engineering, China Academy of Engineering Physics, Mianyang, Sichuan 621999 China; 4 National Laboratory for Infrared Physics, Shanghai Institute of Technical Physics, Chinese Academy of Sciences, Shanghai, 200083 China

**Keywords:** Al_2_O_3_/ZnO nanolaminates, Atomic layer deposition, Morphological properties, Optical properties, Electrical properties

## Abstract

This report mainly focuses on the investigation of morphological, optical, and electrical properties of Al_2_O_3_/ZnO nanolaminates regulated by varying bilayer thicknesses. The growth mechanism of nanolaminates based on atomic layer deposition and Al penetration into ZnO layer are proposed. The surface roughness of Al_2_O_3_/ZnO nanolaminates can be controlled due to the smooth effect of interposed Al_2_O_3_ layers. The thickness, optical constants, and bandgap information of nanolaminates have been investigated by spectroscopic ellipsometry measurement. The band gap and absorption edge have a blue shift with decreasing the bilayer thickness on account of the Burstein-Moss effect, the quantum confinement effect and the characteristic evolution of nanolaminates. Also, the carrier concentrations and resistivities are found to be modified considerably among various bilayer thicknesses. The modulations of these properties are vital for Al_2_O_3_/ZnO nanolaminates to be used as transparent conductor and high resistance layer in optoelectronic applications.

## Background

Nanolaminate is a composite structure formed by different stacking sequences of diverse materials, and the layer thickness is general at the nanometer scale [1–4]. This multilayered structure can endow the nanolaminate with unique properties, and these properties depend on or may be better than those of the constituent materials [5–7]. In recent years, a new kind of materials based on the structure of nanolaminate is began to be utilized for energy storage devices [8], innovative optical elements [9], and temperature sensitive substrates for biosensors [10]. Recently, Viter et al. explored on the tuning of structural properties and the enhancement of electronic and optical properties of 1D PAN (polyacrylonitrile) ZnO/Al_2_O_3_ nanolaminates which will allow applications in different fields such as sensors and biosensors [11]. Baitimirova et al. also investigated the tuning of structural and optical properties of graphene/ZnO nanolaminates which may find applications in optical, bio-, and chemical sensors [12].

As one of the most promising candidates for transparent conductive oxide (TCO) materials, Al-doped ZnO (AZO) film has many advantages, such as abundant resource, low cost, nontoxicity, and good stability in hydrogen plasma. In general investigations, controlling the Al doping level is a common method to improve and modify the optical and electrical behaviors of AZO materials [13, 14], which is crucial to achieve functionalization and tunability of TCO-based devices [15, 16]. However, few reports involve in the performance modulation of AZO by changing the structures of Al_2_O_3_/ZnO nanolaminates which is more simple and effective in semiconductor manufacturing process.

The atomic layer deposition (ALD) technique is suitable for fabricating nanolaminate structures for different purposes and applications [17–19]. This technique is based on self-limiting surface chemical reactions with excellent deposition effect, which can make the thicknesses of individual nanolayers well-controlled for the composite stack. Furthermore, between different sublayers, good nucleation and adhesion can be realized by designing the surface reactions. Therefore, high-quality nanolaminates with uniform and smooth surface can be realized by ALD technique, and the thickness can be controlled accurately as well.

In this work, Al_2_O_3_ and ZnO materials were adopted to realize the nanolaminate structures in order to investigate the tunable characteristics of AZO by changing the bilayer thickness of Al_2_O_3_/ZnO nanolaminates. We investigate their morphological, optical, and electrical properties. The growth mechanism of nanolaminates and Al penetration into ZnO layer are proposed and discussed. With decreasing bilayer Al_2_O_3_/ZnO thickness in the nanolaminates, blue shift of the bandgap is observed and discussed on the basis of the Burstein-Moss (BM) effect, the quantum confinement effect, and the characteristic evolution of the nanolaminates. The tunable electrical properties are exposed by using a measurement system based on the Hall effect. It gives valuable references and ideas that transparent conductor and high-resistance layer can be achieved by varying the bilayer thickness in the nanolaminates.

## Methods

### Synthesis of Nanolaminates by ALD

Al_2_O_3_/ZnO nanolaminates based on Al_2_O_3_-ZnO bilayer stacks were deposited on SiO_2_/Si and quartz substrates by ALD technique. During deposition procedure, the reactor (PICOSUN) temperature was 150 °C. The precursors for Zn, Al, and O were diethylzinc [DEZ; Zn(C_2_H_5_)_2_], trimethylaluminum [TMA; Al (CH_3_)_3_], and deionized water (H_2_O), respectively. The precursor carrier and purge gas was the high purity nitrogen (N_2_, flow rate 50 sccm). It was used to carry precursors into the chamber and bring the needless products out of the chamber.

**Fig. 1:**
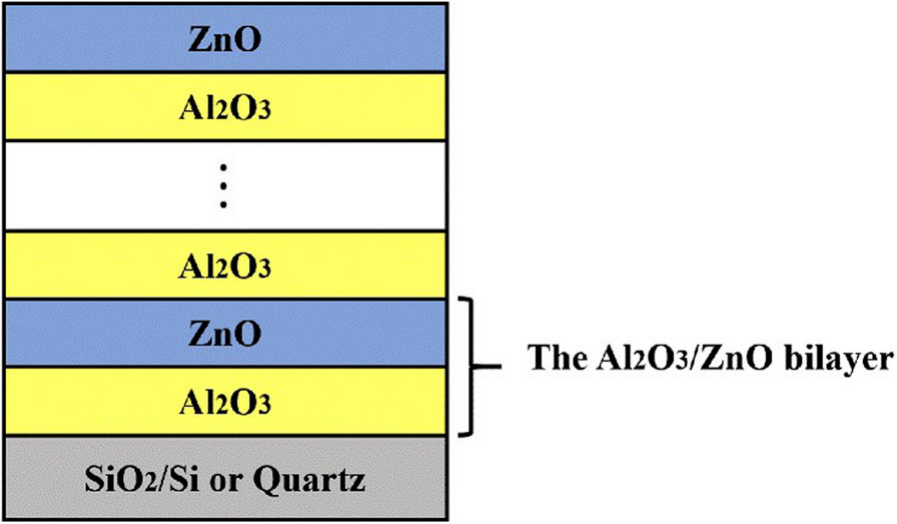
The structure diagram of the Al_2_O_3_/ZnO nanolaminates

In order to grow the Al_2_O_3_ layers, the TMA and H_2_O were alternatively brought into the reactor chamber through TMA-H_2_O cycles (TMA/exposure/N_2_/H_2_O/exposure/N_2_) with pulse time of 0.03/3/15/0.03/5/15 s. The surface reactions of ALD Al_2_O_3_ layers can be defined by two self-limiting reactions as follows [20]:

1 $$ {\mathrm{AlOH}}^{\ast }+\kern0.5em \mathrm{Al}{\left({\mathrm{CH}}_3\right)}_3\to \mathrm{AlOAl}{{\left({\mathrm{CH}}_3\right)}_2}^{\ast }+\kern0.5em {\mathrm{CH}}_4 $$

2 $$ A\mathrm{lOAl}{{\left({\mathrm{CH}}_3\right)}_2}^{\ast }+\kern0.5em {\mathrm{H}}_2\mathrm{O}\to A{\mathrm{lOAlOH}}^{\kern0.5em \ast }+\kern0.5em {\mathrm{CH}}_4 $$

where the asterisks indicate the surface species. As for ZnO layers, DEZ-H_2_O cycles of ZnO were the same as the TMA-H_2_O. The surface reactions of ALD ZnO layers are given by [20]

3 $$ {\mathrm{ZnOH}}^{\kern0.5em \ast }+\kern0.5em \mathrm{Zn}{\left({\mathrm{C}}_2{\mathrm{H}}_5\right)}_2\to {\mathrm{ZnOZnC}}_2{{\mathrm{H}}_5}^{\ast }+\kern0.5em {\mathrm{C}}_2{\mathrm{H}}_6 $$

4 $$ {\mathrm{ZnOZnC}}_2{{\mathrm{H}}_5}^{\ast }+\kern0.5em {\mathrm{H}}_2\mathrm{O}\to {\mathrm{ZnOZnOH}}^{\ast }+\kern0.5em {\mathrm{C}}_2{\mathrm{H}}_6 $$

where the asterisks also indicate the surface species. The structure diagram of ALD Al_2_O_3_/ZnO nanolaminates is shown in Fig. 1. For all nanolaminates, the interface with the substrate was Al_2_O_3_, while ZnO was the top layer at the surface of nanolaminates. The bilayer is constructed by two individual layers, i.e., Al_2_O_3_ and ZnO, with the same thickness. In order to guarantee the same thicknesses of total nanolaminates, the number of bilayer was increased with the decrease of bilayer thickness. So five kinds of samples were prepared, named as 2 (25/25 nm), 5 (10/10 nm), 10 (5/5 nm), 25 (2/2 nm), and 50 (1/1 nm). The details can be found in Table 1. Note that the parameters in Table 1 are the empirical values, which are summarized from our preliminary experiments.

**Table 1:** The parameters of samples with different bilayer thicknesses

Sample	Layer thickness (nm)		Cycles		The number of bilayer
	Al_2_O_3_	ZnO	Al_2_O_3_	ZnO	
2 (25/25 nm)	25	25	250	150	2
5 (10/10 nm)	10	10	100	60	5
10 (5/5 nm)	5	5	50	30	10
25 (2/2 nm)	2	2	20	12	25
50 (1/1 nm)	1	1	10	6	50

### Characterization

The morphological characterization of Al_2_O_3_/ZnO nanolaminates was carried out by transmission electron microscope (TEM; FEI Tecnai G2 F20) and atomic force microscopy (AFM; Bruker Dimension Icon VT-1000, Santa Barbara, CA). The thickness, optical constants, and bandgap information were determined by spectroscopic ellipsometry (SE; J.A. Woollam, Inc., M2000X-FB-300XTF) measurements in the wavelength range of 200–1000 nm under an incident angle of 65°. The optical transmittance of nanolaminates has also been studied in the wavelength range of 200–1000 nm by using a dual beam spectrophotometer (Shimadzu UV-3600). A Hall effect measurement system (Ecopia HMS3000) was used to obtain electrical properties of the samples with a four-point probe.

## Results and Discussion

### Morphological Characteristics

**Fig. 2:**
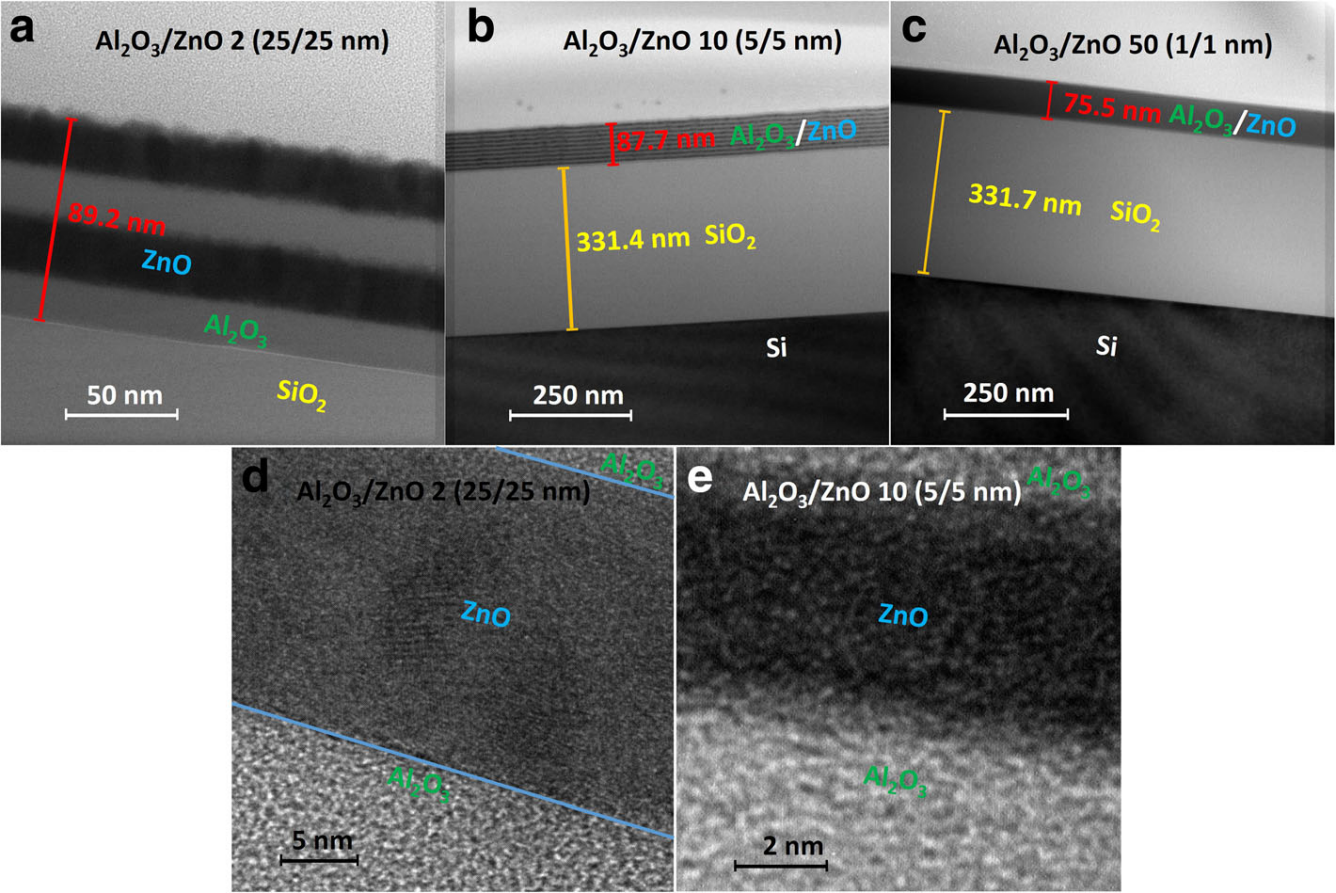
TEM images of Al_2_O_3_/ZnO nanolaminates with different bilayer thicknesses: **a** 50 nm, **b** 10 nm, and **c** 2 nm. And high-magnification images: **d** 50 nm and **e** 10 nm

Nanolaminates with different bilayer thicknesses grown on SiO_2_/Si substrates were measured on cross sections with TEM. Three illustrative examples of nanolaminates with a bilayer thickness of 50, 10, and 2 nm are shown in Fig. 2, including the high-magnification images of 50- and 10-nm samples. Clear boundaries can be observed between Al_2_O_3_ and ZnO layers, and the thickness of total nanolaminates is indicated. With the help of X-ray diffraction (Bruker D8 ADVANCE) measurements in advance (not given here), we find no characteristic peaks of Al_2_O_3_ and ZnO, and hence that all of the as-grown nanolaminates have amorphous state. This statement can be verified by the high-magnification TEM images. Even though Al_2_O_3_/ZnO 2 (25/25 nm) samples have the thickest bilayer in this work, the crystallization process does not exist in them.

As reported elsewhere [6, 21], the Al_2_O_3_ layer in Al_2_O_3_/ZnO nanolaminates prepared by ALD method is always in amorphous phase and it can block the ZnO crystal growth because the ZnO is forced to renucleate on the Al_2_O_3_ surface. The crystallization process of ultrathin layers is very complex, and many factors should be taken into account, such as the interface energies, the thickness of the layers, the melting point of the system, and the bulk amorphous crystallization temperature [6, 22]. Viter et al. discovered that Al_2_O_3_/ZnO nanolaminates with bilayer thickness of 20 nm (ratio 1:1) have amorphous nature and they attributed this result to the minimum thickness required to allow crystallization [22]. López et al. found similar phenomenon and they thought that the pulse as well as the purge duration of the growth procedure was too short to give their films enough time for generating some ordering and some crystalline phases [23]. Meanwhile, the Bohr radius of bulk ZnO is 23 Å [4]. Al_2_O_3_/ZnO 25 (2/2 nm) and Al_2_O_3_/ZnO 50 (1/1 nm) nanolaminates have ZnO thicknesses smaller than the Bohr radius; therefore, the quantum confinement effect should be taken into consideration. Especially for semiconducting sublayers ZnO, it is believed that this effect can cause dramatic change in the dielectric behavior [21], and we will discuss it in the following content.

**Fig. 3:**
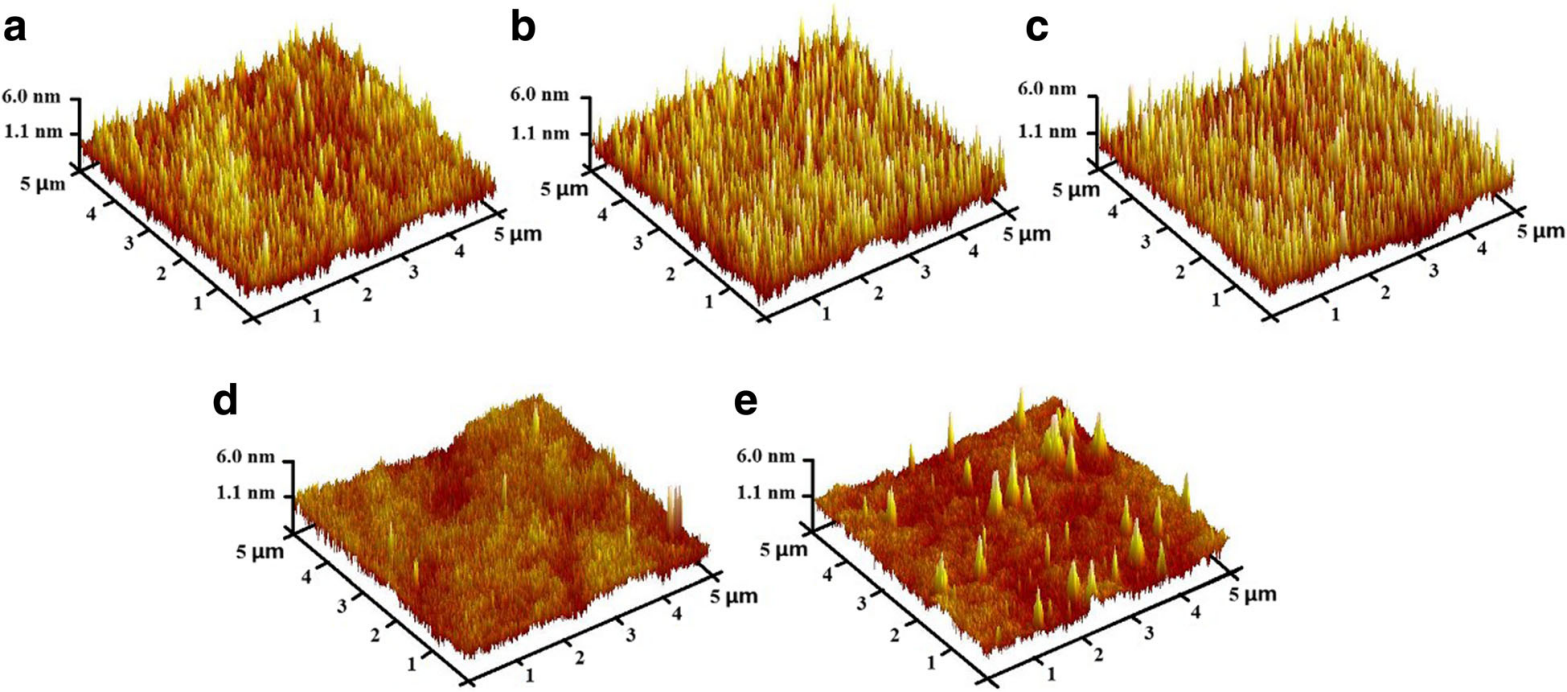
AFM 3D images of nanolaminates with different bilayer thicknesses: **a** 2 (25/25 nm), **b** 5 (10/10 nm), **c** 10 (5/5 nm), **d** 25 (2/2 nm), and **e** 50 (1/1 nm)

**Fig. 4:**
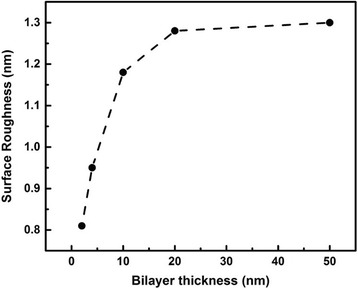
Surface roughness of nanolaminates with different bilayer thicknesses

To investigate the surface morphologies of the nanolaminates, AFM measurement is applied for the samples deposited on SiO_2_/Si substrates, and the 3D results are shown in Fig. 3. It can be observed that the hill-shaped features are dominated on the sample surface and surface height decreases with lower bilayer thickness. Samples with low bilayer thicknesses, i.e., Al_2_O_3_/ZnO 25 (2/2 nm) and Al_2_O_3_/ZnO 50 (1/1 nm), show smooth surface with insignificant surface roughness. The root-mean-square roughness *R*_q_ of each nanolaminate is estimated from AFM data and approximately ranges from 0.81 to 1.30 nm. Moreover, the relationship between bilayer thickness and *R*_q_ is revealed in Fig. 4. At first, the values of *R*_q_ show linear behavior vs. the increase of bilayer thickness, then it remains stable when the bilayer thickness increases to a certain value, as is the case for other studies [23, 24]. The Al_2_O_3_ in this work is in amorphous phase under the above growth condition, which has been proved in our previous report as well [25]. The amorphous Al_2_O_3_ layer is very smooth and conforms to the topography of underlying ZnO layers [26]. As mentioned above, due to the interposed Al_2_O_3_ layer, the crystal growth of ZnO is consequently interrupted. Through restricting the size of the ZnO nanocrystals, the interposed Al_2_O_3_ layers prevent the Al_2_O_3_/ZnO nanolaminates from roughening [24]. This smooth effect has been proven to have little to do with the Al_2_O_3_ layer thickness and only relates to the number of interposed Al_2_O_3_ layers [24]. Therefore, with the decrease of bilayer thickness, more Al_2_O_3_ layers were interposed into the nanolaminates to smooth roughness, which leads to the nanolaminates more smooth. When the bilayer thickness increases to a certain value, this smooth effect is no longer obvious.

### Optical Properties

**Fig. 5:**
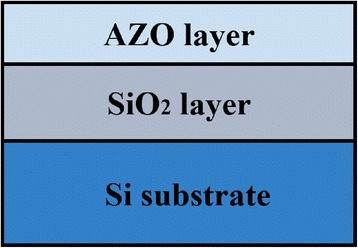
Optical model of samples grown on SiO_2_/Si substrate for SE analysis

By performing SE measurement [17, 27, 28] which is based on recording and calculating the change of a reflected linearly polarized light from the surface of samples, the optical constants and film thickness of nanolaminates can be deduced from the raw data. In order to get more accurate details, the nanolaminates grown on SiO_2_/Si substrates are chosen as the testing object because of its opaque to light during SE measurements. After raw data acquisition, a multilayer model is constructed containing semi-infinite Si substrate, SiO_2_ layer, and AZO layer, as revealed in Fig. 5. The nanolaminates, i.e., the AZO layer in the model, are considered as a whole to be fitted. Oxidation layer of Si substrate is about 330 nm, which is directly substituted into the model without fitting. Moreover, no Bruggeman effective media approximation is introduced in this optical model because of the ignorable surface roughnesses of samples based on the AFM results. On account of this optical model, the Forouhi-Bloomer (FB) dispersion model is used to fit the ellipsometry spectra (*Ψ* and Δ in the range of 200–1000 nm) of the nanolaminates [29, 30]. The final thickness and optical properties are fitted and evaluated to minimize the root-mean-square error (RMSE) which follows:

5 $$ \mathrm{RMSE}=\sqrt{\frac{1}{2N-M-1}{\sum}_{i=1}^N\left[{\left({\psi}_i^{\mathrm{cal}}-{\psi}_i^{\mathrm{exp}}\right)}^2+{\left({\varDelta}_i^{\mathrm{cal}}-{\varDelta}_i^{\mathrm{exp}}\right)}^2\right]} $$

Here, *N*, *M*, exp, and cal represent the number of data points in the spectra, the number of variable parameters in the model, the experimental data, and the calculated data, respectively.

The fitted thicknesses of nanolaminates are shown in Table 2. They are very close to the values obtained from TEM measurements, indicating the accuracy of fitting process. The fitting error RMSE is also revealed in Table 2, and the value is within permitted, demonstrating the reliability of fitting results. The thicknesses of sample 2 (25/25 nm), 5 (10/10 nm), and 10 (5/5 nm) show a smooth trend, and the small fluctuation results from varying degrees of ALD surface chemical reactions. In contrast, the thicknesses of sample 25 (2/2 nm) and 50 (1/1 nm) decline obviously with decreasing bilayer thickness. It follows that the Al_2_O_3_/ZnO nanolaminates show different thickness with low bilayer thickness, even if the cycle number stays the same in deposition process. Table 3 summarizes the growth rates of Al_2_O_3_ and ZnO sublayers (thickness ratio 1:1) using the thicknesses listed in Table 2. The values increase at first and saturate finally when the cycles in sublayers increase. The variation in film thickness and growth rate may result from the interfacial reaction between Al_2_O_3_ and ZnO layers which will be introduced in the following content, and samples with lower bilayer thickness will be more affected. Karvonen et al. gave similar explanation, and they attributed the variation in growth rate to the TMA etching of ZnO during the Al_2_O_3_ growth [7]. Elam et al. found that the growth rates of Al_2_O_3_ and ZnO increase with the number of ALD cycles [24]. They concluded that the reduced growth rate of early ALD cycles may result from the nucleation process occurring when making the transition from Al_2_O_3_ to ZnO and from ZnO to Al_2_O_3_. Only when new crystals are formed does the growth rate achieve the steady state value.

**Table 2:** The thicknesses of AZO layer obtained from TEM and fitted by SE, and the fitting error RMSE

Sample	Thickness_TEM_ (nm)	Thickness_SE_ (nm)	RMSE
2 (25/25 nm)	89.2	86.64	1.17
5 (10/10 nm)	–	88.18	0.83
10 (5/5 nm)	87.7	87.31	0.77
25 (2/2 nm)	–	80.90	0.69
50 (1/1 nm)	75.5	74.80	0.42

**Table 3:** The growth rates of samples with different bilayer thicknesses

Sample	Growth rate (Å/cycle) cycles			
	Al_2_O_3TEM_	ZnO_TEM_	Al_2_O_3SE_	ZnO_SE_
2 (25/25 nm)	0.89	1.49	0.87	1.44
5 (10/10 nm)	–	–	0.88	1.47
10 (5/5 nm)	0.88	1.46	0.87	1.46
25 (2/2 nm)	–	–	0.81	1.35
50 (1/1 nm)	0.76	1.26	0.75	1.25

**Fig. 6:**
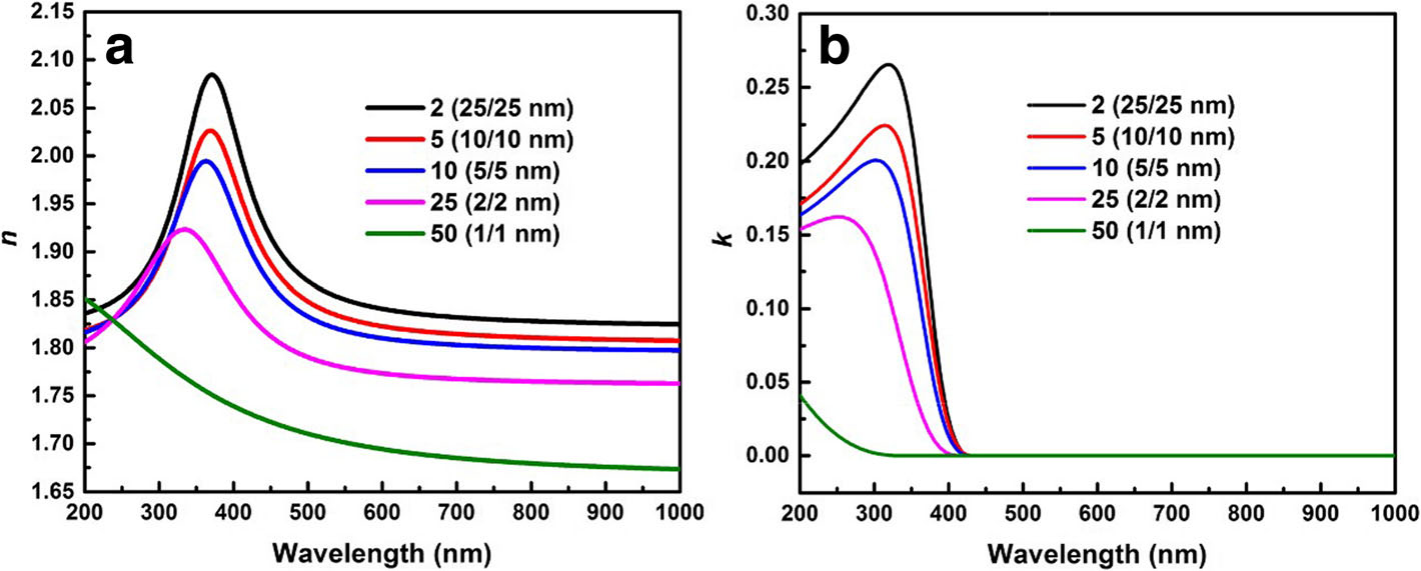
The optical constants of nanolaminates grown on SiO_2_/Si substrate. **a** The refractive index *n*. **b** The extinction coefficient *k*

The optical constants of Al_2_O_3_/ZnO nanolaminates are illustrated in Fig. 6. It shows various refractive index *n* and extinction coefficient *k* with different bilayer thicknesses. Figure 6a describes the refractive index dispersion spectra of the nanolaminates with different bilayer thicknesses. The values of *n* decrease gradually with decline of bilayer thickness in the range of 50 to 2 nm due to the growth change and the Al penetration [21, 31]. The *n*(*λ*) characteristic of ZnO can be observed for nanolaminates with bilayer thicknesses of 50, 20, and 10 nm. And this line shape slowly degenerates and disappears when the bilayer thickness is below 4 nm. Consequently, the *n*(*λ*) characteristics tend to behaving like Al_2_O_3_ as the sample 50 (1/1 nm) shown. The *k* dispersion spectra can be found in Fig. 6b. Different curves represent different samples with various bilayer thicknesses. In the region of 430–1000 nm, the extinction coefficients are approximately equal to 0, i.e., the nanolaminates are almost transparent in that wavelength region. Meanwhile, a blue shift occurs at the absorption edge with decreasing bilayer thickness. The shift distance of sample 25 (2/2 nm) and 50 (1/1 nm) is larger, so the absorption edge gradually moves out of the spectral region and presents the characteristics of Al_2_O_3_. As a whole, the characteristics of optical constants transfer from ZnO to Al_2_O_3_. The observed changes of *n* and *k* could be determined by two physical phenomena. On the one hand, they are affected by the quantum confinement effect. We can see that samples 25 (2/2 nm) and 50 (1/1 nm) have sublayer thicknesses smaller than the Bohr radius of bulk ZnO, so their dielectric behaviors change more dramatically than the other samples. On the other hand, it is based on the growth mechanism which leads to Al penetration into ZnO layers [22, 24]. According to the growth mechanism, the substitution reaction of Zn with Al may occur in the interface between ZnO and Al_2_O_3_ layers:

6 $$ \mathrm{Zn}\hbox{-} {\mathrm{OH}}^S+\kern0.5em \mathrm{Al}{{\left({\mathrm{C}}_2{\mathrm{H}}_5\right)}_3}^g\uparrow \to \mathrm{AlOH}\hbox{-} {\mathrm{C}}_2{{\mathrm{H}}_5}^s+\kern0.5em \mathrm{Zn}{{\left({\mathrm{C}}_2{\mathrm{H}}_5\right)}_2}^g\uparrow $$

where ZnO-OH and Al (C_2_H_5_)_3_ are the substance on the surface and gas phase, correspondently. Because of this interfacial reaction, Al doping into ZnO layers may happen and ZnO ratio in nanolaminates can be reduced. Therefore, with the decrease of the bilayer thickness, the interface between ZnO and Al_2_O_3_ layers increases, and the ratio of ZnO in the nanolaminates decreases accordingly. This can be verified by the high-magnification TEM images shown in Fig. 2d, e. When the bilayer thickness decreases, the boundaries between Al_2_O_3_ and ZnO layers become wider and blurrier. It makes the characteristic of whole nanolaminates transfers to that of Al_2_O_3_.

**Fig. 7:**
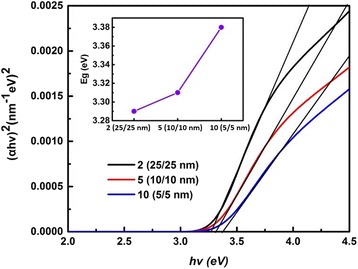
Evaluated optical bandgap of nanolaminates with different bilayer thicknesses

For better understanding of the blue shift of absorption edge, Tauc extrapolation is applied to evaluate the bandgap information of the nanolaminates with a bilayer thickness of 50, 20, and 10 nm. To evaluate the bandgap energies, extinction coefficients of the nanolaminates were used. Extinction, bandgap energy, and absorption coefficients are associated according to the following formulas [32]:

7 $$ {\left(\alpha h\upsilon \right)}^2=A\left(E-{E}_g\right) $$

8 $$ \alpha =\frac{4 k\pi}{\lambda } $$

where *α* is the optical absorption coefficient, *A* is a constant, and *E*_*g*_ is the optical bandgap energy. On the basis of Eqs. (7) and (8), a plot of (*αhν*)^2^ vs. *hν* has been made as demonstrated in Fig. 7. The value of bandgap energy *E*_*g*_ can be graphically determined by *x* axis and the linear fitting in linear part of the absorption edge, which is provided in the inset figure of Fig. 7. The bandgap information of samples 25 (2/2 nm) and 50 (1/1 nm) is not revealed in Fig. 7, because the linear part of the absorption edge exceeds the spectral range evolved from the spectra of extinction coefficient, which could lead to inaccurate results. From Fig. 7, it can be seen that the bandgap energy of nanolaminate displays a growing trend with decreasing bilayer thickness, which could be interpreted by the BM effect [33–35]. Interposed Al^3+^ takes the place of Zn^2+^ in the interface of Al_2_O_3_/ZnO layers and provides an extra electron. So in nanolaminates, the concentration of free carriers increases, causing the bandgap energy moves to higher energy region. The following equation can describe this effect exactly [35]:

9 $$ {E}_g={E}_g^0+\varDelta {E}_g^{\mathrm{BM}}={E}_g^0+\frac{h^2}{8{m}_e^{\ast }}{\left(\frac{3}{\pi}\right)}^{2/3}{n}_e^{2/3} $$

where Δ*E*_*g*_^BM^ and *E*_*g*_^*0*^ represent the increment of bandgap caused by BM effect and intrinsic forbidden bandwidth, while *h*, *m*_e_^*^, and *n*_*e*_ are the Plank’s constant, effective electron mass in the conduction band, and electron carrier density, respectively.

**Fig. 8:**
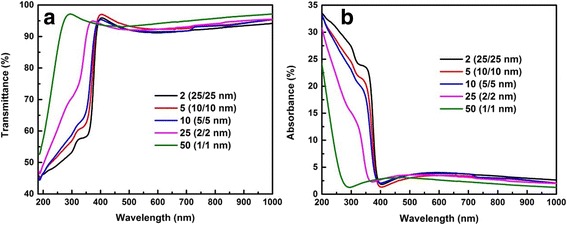
**a** Transmittance and **b** absorbance spectra of nanolaminates grown on quartz substrates with different bilayer thicknesses

Figure 8 gives the spectra of transmittance and absorbance of the whole group. It can be found that sharp absorption edges are located in the region from 200 to 400 nm, namely ultraviolet region. Importantly, absorption edges move to the shorter wavelength (blue shift) with decreasing bilayer thickness, and this trend is exactly close to previous results calculated from SE measurement. This blue shift is due to the BM effect that makes the increase of bandgap. However, the blue shift is not successional, because in the nanolaminates of 25 (2/2 nm) and 50 (1/1 nm), the quantum confinement effect becomes dominant, and the interfacial reaction intensifies which makes the nanolaminates show the characteristics of Al_2_O_3_ gradually. At this point, the blue shift is the total contribution of the BM effect, the quantum confinement effect, and the characteristic evolution of nanolaminates. That is to say, these three factors cause the enormous shift of the absorption edge. As a whole, the absorption edge can be modulated by the bilayer thickness in the ultraviolet region (200–400 nm). According to this, it can be applied as ultraviolet detector. Besides, all of the Al_2_O_3_/ZnO nanolaminates show a transmittance above 90% in visible and near-infrared region, along with a sharp absorption band edge. The transmittance here shows nearly similar value and trend with that of many other TCO materials [36], which makes it possible to be applied as TCO material.

### Electrical Properties

**Fig. 9:**
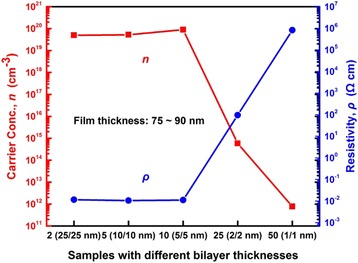
Carrier concentration and resistivity of nanolaminates grown on quartz substrate with different bilayer thicknesses

The Hall effect measurement is conducted to correlate the analyses with the electrical properties of the Al_2_O_3_/ZnO nanolaminates. Nanolaminates prepared on quartz substrates are selected as the test samples to remove the spatial resistivity distribution, and Fig. 9 displays the testing results. At the beginning, the carrier concentration and resistivity show little change and remain at around 10^19^ cm^−3^ and 10^−2^Ω cm, respectively. With decreasing bilayer thickness, the carrier concentration sharply drops and the resistivity increases as well. It can be interpreted by the interfacial reaction of Al_2_O_3_/ZnO layers which results in the characteristic evolution of nanolaminates. The nanolaminates show insulation characteristic of Al_2_O_3_ gradually and realize the tunableness of resistivity by changing their bilayer thickness. In addition, the values of the carrier concentration of nanolaminates 2 (25/25 nm), 5 (10/10 nm), and 10 (5/5 nm) are 4.99 × 10^19^, 5.26 × 10^19^, and 8.91 × 10^19^ cm^−3^, respectively. It shows a slow growth in accordance with the explanation of the bandgap results, and the values approximately equal to those of TCO materials from the results of other reports [25, 37]. So these three kinds of nanolaminates possess not only favorable electrical conductivity but also excellent light transmittance in the visible and near-infrared region. It is vital for Al_2_O_3_/ZnO nanolaminates to play a role in the field of transparent conductor. The samples 25 (2/2 nm) and 50 (1/1 nm) present insulation characteristic and realize the tunableness of resistivity, which can be applied as high-resistivity layer in semiconductor devices.

## Conclusions

We have investigated the morphological, optical, and electrical properties of Al_2_O_3_/ZnO nanolaminates among various bilayer thicknesses ranging from 2 to 50 nm. The clear layer boundaries and low surface roughness show high-quality morphologies of nanolaminates prepared by ALD method. With the decrease of the bilayer thickness, inserted Al_2_O_3_ layers in nanolaminates begin to limit the roughness, which leads to the nanolaminates more smooth. When the bilayer thickness reaches a certain value, this roughness limitation can be ignored. The thickness, optical constants, and bandgap information of nanolaminates have been extracted from SE analysis. With decreasing bilayer thickness, the absorption edge of extinction coefficient has a blue shift, and the optical band gap energies show a growing trend, because the BM effect, the quantum confinement effect, and the characteristic evolution of nanolaminates have significant influence on them. This blue shift also occurs in the transmission and absorbance spectra with high transmittance beyond 90% in the visible and near-infrared region. Moreover, by varying the bilayer thickness, the electrical properties also show two kinds of characteristics, and the modulation of characteristics is realized. The nanolaminates 2 (25/25 nm), 5 (10/10 nm), and 10 (5/5 nm) show high carrier concentration above 10^19^ cm^−3^, which can be applied as transparent conductive material. And also, the nanolaminates 25 (2/2 nm) and 50 (1/1 nm) possessing high resistivity can be used as high-resistivity layer in semiconductor manufacturing process.

## Abbreviations

AFM: Atomic force microscopy
ALD: Atomic layer deposition
AZO: Al-doped ZnO
BM: Burstein-Moss
DEZ: Diethylzinc
FB: Forouhi-Bloomer
RMSE: Root-mean-square error
SE: Spectroscopic ellipsometry
TCO: Transparent conductive oxide
TEM: Transmission electron microscope
TMA: Trimethylaluminum

## References

[1] Rowlette PC, Wolden CA (2010). Pulsed plasma-enhanced chemical vapor deposition of Al_2_O_3_–TiO_2_ nanolaminates. Thin Solid Films.

[2] Seo SW, Jung E, Chae H, Cho SM (2012). Optimization of Al_2_O_3_/ZrO_2_ nanolaminate structure for thin-film encapsulation of OLEDs. Org Electron.

[3] Richardson JJ, Björnmalm M, Caruso F (2015). Multilayer assembly. Technology-driven layer-by-layer assembly of nanofilms. Science.

[4] Li J, Bi X (2016). Temperature- and frequency-dependent dielectric behaviors of insulator/semiconductor (Al_2_O_3_/ZnO) nanolaminates with various ZnO thicknesses. J Phys D Appl Phys.

[5] Lackner JM, Waldhauser W, Major R, Hartmann P (2013). Biomimetics in thin film design—wrinkling and fracture of pulsed laser deposited films in comparison to human skin. Surf Coat Technol.

[6] Raghavan R, Bechelany M, Parlinska M, Frey D (2012). Nanocrystalline-to-amorphous transition in nanolaminates grown by low temperature atomic layer deposition and related mechanical properties. Appl Phys Lett.

[7] Karvonen L, Saynatjoki A, Chen Y, Jussila H, Ronn J, Ruoho M, Alasaarela T, Kujala S, Norwood RA, Peyghambarian N (2013). Enhancement of the third-order optical nonlinearity in ZnO/Al_2_O_3_ nanolaminates fabricated by atomic layer deposition. Appl Phys Lett.

[8] Elias J, Bechelany M, Utke I, Erni R, Hosseini D, Michler J, Philippe L (2012). Urchin-inspired zinc oxide as building blocks for nanostructured solar cells. Nano Energy.

[9] Allan-Wojtas P, Hildebrand PD, Braun PG, Smith-King HL, Carbyn S, Renderos WE (2011). Highly active oxide photocathode for photoelectrochemical water reduction. Nat Mater.

[10] Jędrzejewska-Szczerska M, Wierzba P, Chaaya AA, Bechelany M, Miele P, Viter R, Mazikowski A, Karpienko K, Wróbel M (2015). ALD thin ZnO layer as an active medium in a fiber-optic Fabry–Perot interferometer. Sensor Actuat A-Phys.

[11] Viter R, Iatsunskyi I, Fedorenko V, Tumenas S, Balevicius Z, Ramanavicius A, Balme S, Kempiński M, Nowaczyk G, Jurga S, Bechelany M (2016). Enhancement of electronic and optical properties of ZnO/Al_2_O_3_ nanolaminates coated electrospun nanofibers. J Phys Chem C.

[12] Baitimirova M, Viter R, Andzane J, Lee A, Voiry D, Iatsunskyi I, Coy E, Mikoliunaite L, Tumenas S, Zaleski K, Balevicius Z, Baleviciute I, Ramanaviciene A, Ramanavicius A, Jurga S, Erts D, Bechelany M (2016). Tuning of structural and optical properties of graphene/ZnO nanolaminates. J Phys Chem C.

[13] Benhaoua B, Rahal A, Benramache S (2014). The structural, optical and electrical properties of nanocrystalline ZnO:Al thin films. Superlattices Microst.

[14] Lee DJ, Kim HM, Kwon JY, Choi H, Kim SH, Kim KB (2011). Structural and electrical properties of atomic layer deposited Al-doped ZnO films. Adv Funct Mater.

[15] Schulze K, Maennig B, Leo K, Tomita Y (2007). Organic solar cells on indium tin oxide and aluminum doped zinc oxide anodes. Appl Phys Lett.

[16] Meyer J, Hamwi S, Johannes HH, Riedl T, Kowalsky W (2008). Indium-free transparent organic light emitting diodes with Al doped ZnO electrodes grown by atomic layer and pulsed laser deposition. Appl Phys Lett.

[17] Zheng H, Zhang RJ, Xu JP, Wang S, Zhang T, Sun Y, Zheng YX, Wang SY, Chen X, Chen LY (2016). Thickness-dependent optical constants and annealed phase transitions of ultrathin ZnO films. J Phys Chem C.

[18] Kim LH, Jeong YJ, An TK, Park S, Jang JH, Nam S, Jang J, Kim SH, Park CE (2015). Optimization of Al_2_O_3_/TiO_2_ nanolaminate thin films prepared with different oxide ratios, for use in organic light-emitting diode encapsulation, via plasma-enhanced atomic layer deposition. Phys Chem Chem Phys.

[19] Marichy C, Bechelany M, Pinna N (2012). ChemInform abstract: atomic layer deposition of nanostructured materials for energy and environmental applications. Adv Mater.

[20] Na JS, Peng Q, Scarel G, Parsons GN (2009). Role of gas doping sequence in surface reactions and dopant incorporation during atomic layer deposition of Al-doped ZnO. Chem Mater.

[21] Chaaya AA, Viter R, Baleviciute I, Bechelany M, Ramanavicius A, Gertnere Z, Erts D, Smyntyna V, Miele P (2016). Tuning optical properties of Al_2_O_3_/ZnO nanolaminates synthesized by atomic layer deposition. J Phys Chem C.

[22] Viter R, Baleviciute I, Chaaya AA, Mikoliunaite L, Balevicius Z, Ramanavicius A, Zalesska A, Vataman V, Smyntyna V, Gertnere Z (2015). Optical properties of ultrathin Al_2_O_3_/ZnO nanolaminates. Thin Solid Films.

[23] López J, Martínez J, Abundiz N, Domínguez D, Murillo E, Castillón FF, Machorro R, Farías MH, Tiznado H (2016). Thickness effect on the optical and morphological properties in Al_2_O_3_/ZnO nanolaminate thin films prepared by atomic layer deposition. Superlattices Microst.

[24] Elam JW, Sechrist ZA, George SM (2002). ZnO/Al_2_O_3_ nanolaminates fabricated by atomic layer deposition: growth and surface roughness measurements. Thin Solid Films.

[25] Zhai CH, Zhang RJ, Chen X, Zheng YX, Wang SY, Liu J, Dai N, Chen LY (2016). Effects of Al doping on the properties of ZnO thin films deposited by atomic layer deposition. Nanoscale Res Lett.

[26] Ott AW, Klaus JW, Johnson JM, George SM (2009). Erratum to “Al_2_O_3_ thin film growth on Si (100) using binary reaction sequence chemistry”. Thin Solid Films.

[27] Xu JP, Zhang RJ, Zhang Y, Wang ZY, Chen L, Huang QH, Lu HL, Wang SY, Zheng YX, Chen LY (2016). The thickness-dependent band gap and defect features of ultrathin ZrO2 films studied by spectroscopic ellipsometry. Phys Chem Chem Phys.

[28] Li DH, Zheng H, Wang ZY, Zhang RJ, Zhang H, Zheng YX, Wang XY, Zhang W, Chen LY (2017). Dielectric functions and critical points of crystalline WS_2_ ultrathin films with tunable thickness. Phys Chem Chem Phys.

[29] Forouhi AR, Bloomer II (1986). Optical dispersion relations for amorphous semiconductors and amorphous dielectrics. Phys Rev B.

[30] Forouhi AR, Bloomer II (1986). Optical properties of crystalline semiconductors and dielectrics. Phys Rev B.

[31] López J, Solorio E, Borbón-Nuñez HA, Castillón FF, Machorro R, Nedev N, Farías MH, Tiznado H (2017). Refractive index and bandgap variation in Al_2_O_3_-ZnO ultrathin multilayers prepared by atomic layer deposition. J Alloys Compd.

[32] Li QH, Zhu D, Liu W, Liu Y, Ma XC (2008). Optical properties of Al-doped ZnO thin films by ellipsometry. Appl Surf Sci.

[33] Fujiwara H, Kondo M (2005). Effects of carrier concentration on the dielectric function of ZnO:Ga and In_2_O_3_:Sn studied by spectroscopic ellipsometry: analysis of free-carrier and band-edge absorption. Phys Rev B.

[34] Burstein E (1954). Anomalous optical absorption limit in InSb. Phys Rev.

[35] Liu Y, Li Q, Shao H (2009). Optical and photoluminescent properties of Al-doped zinc oxide thin films by pulsed laser deposition. J Alloys Compd.

[36] Mendezgamboa JA, Castrorodriguez R, Perezquintana IV, Medinaesquivel RA, Martelarbelo A (2016). A figure of merit to evaluate transparent conductor oxides for solar cells using photonic flux density. Thin Solid Films.

[37] Pradhan AK, Mundle RM, Santiago K, Skuza JR, Xiao B, Song KD, Bahoura M, Cheaito R, Hopkins PE (2014). Extreme tunability in aluminum doped zinc oxide plasmonic materials for near-infrared applications. Sci Rep.

